# Identification of an essential virulence gene of cyprinid herpesvirus 3

**DOI:** 10.1016/j.antiviral.2017.07.002

**Published:** 2017-09

**Authors:** Maxime Boutier, Yuan Gao, Catherine Vancsok, Nicolás M. Suárez, Andrew J. Davison, Alain Vanderplasschen

**Affiliations:** a Immunology-Vaccinology, Department of Infectious and Parasitic Diseases, Fundamental and Applied Research for Animals & Health (FARAH), Faculty of Veterinary Medicine, University of Liège, Liège, Belgium; b MRC-University of Glasgow Centre for Virus Research, Glasgow, United Kingdom

**Keywords:** Herpesvirus, Alloherpesvirus, Cyprinivirus, Cyprinid herpesvirus 3, Koi herpesvirus, Attenuated vaccine

## Abstract

The genus *Cyprinivirus* consists of a growing list of phylogenetically related viruses, some of which cause severe economic losses to the aquaculture industry. The archetypal member, cyprinid herpesvirus 3 (CyHV-3) causes mass mortalities worldwide in koi and common carp. A CyHV-3 mutant was described previously that is attenuated *in vivo* by a deletion affecting two genes (ORF56 and ORF57). The relative contributions of ORF56 and ORF57 to the safety and efficacy profile of this vaccine candidate have now been assessed by analysing viruses individually deleted for ORF56 or ORF57. Inoculation of these viruses into carp demonstrated that the absence of ORF56 did not affect virulence, whereas the absence of ORF57 led to an attenuation comparable to, though slightly less than, that of the doubly deleted virus. To demonstrate further the role of ORF57 as a key virulence factor, a mutant retaining the ORF57 region but unable to express the ORF57 protein was produced by inserting multiple in-frame stop codons into the coding region. Analysis of this virus *in vivo* revealed a safety and efficacy profile comparable to that of the doubly deleted virus. These findings show that ORF57 encodes an essential CyHV-3 virulence factor. They also indicate that ORF57 orthologues in other cypriniviruses may offer promising targets for the rational design of attenuated recombinant vaccines.

## Highlights

• Cyprinid herpesvirus 3 (CyHV-3) causes a lethal disease in common and koi carp and is the archetypal fish alloherpesvirus.
• CyHV-3 ORF57 encodes an essential virulence factor and ORF57 deleted viruses represent attenuated vaccine candidates.
• ORF57 orthologues in other alloherpesviruses may offer promising targets for the design of attenuated recombinant vaccines.

## Graphical abstract

**Figure:**
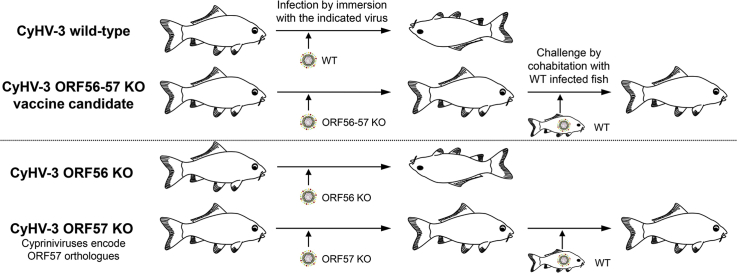

## 1 Introduction

The *Alloherpesviridae* is one of three families in the order *Herpesvirales*, and consists of herpesviruses that infect fish or amphibians (Boutier et al., 2015a). It currently contains 12 species distributed into four genera. One of these genera, *Cyprinivirus*, comprises a growing list (Doszpoly et al., 2015) of phylogenetically related viruses, some of which cause severe economic losses to the aquaculture industry. These include cyprinid herpesvirus 2 (CyHV-2; also known as goldfish hematopoietic necrosis virus), which causes a lethal disease in goldfish (*Carassius auratus*) and gibel carp (*C. gibelio*) (Xu et al., 2013, Xu et al., 2014, Ito and Maeno, 2014), anguillid herpesvirus 1 (AngHV-1), which is responsible for mortalities of up to 30% in cultured and wild European and Japanese eel populations (*Anguilla anguilla* and *A. japonica*) (van Beurden et al., 2012), and cyprinid herpesvirus 3 (CyHV-3; also known as koi herpesvirus), which is the etiological agent of a lethal disease in common carp (*Cyprinus carpio*) and koi carp (Adamek et al., 2014, Rakus et al., 2013).

CyHV-3 is considered to be the archetypal fish alloherpesvirus (Boutier et al., 2015a). Since its emergence in the 1990s, this virus has caused severe economic losses to the carp culture industry worldwide (Adamek et al., 2014, Rakus et al., 2013). For example, outbreaks of CyHV-3 that occurred in Indonesia in 2002 and 2003 were estimated to have resulted in economic losses amounting to US$15 million (Bondad-Reantaso et al., 2005, Sunarto et al., 2005). CyHV-3 is also provoking a societal impact in developing countries by affecting familial aquaculture and thereby limiting access to one of the cheapest sources of animal protein (Boutier et al., 2015a). In addition, it is having an ecological impact by inducing carp mortalities in natural habitats (Ito et al., 2014, Uchii et al., 2013).

Various types of vaccine candidate against CyHV-3 have been developed (Boutier et al., 2015b, O'Connor et al., 2014, Perelberg et al., 2008). Among these, attenuated vaccines seem the most promising because they are more likely to suit practical field constraints, such as the need for mass vaccination of fish weighing only a few grams each. Also, scientific advances are increasingly facilitating the rational design of attenuated vaccines (Rueckert and Guzman, 2012), including deleting genes from a viral genome in a precise way that precludes reversion to virulence (Meeusen et al., 2007). This approach has been taken for CyHV-3 by targeting various open reading frame (ORFs), including ORF16, ORF55, ORF123 and ORF134, which encode a G protein-coupled receptor (GPCR), thymidine kinase (TK), deoxyuridine triphosphatase (dUTPase) and interleukin-10, respectively. However, none of the recombinants lacking these genes has been shown to have a safety and efficacy profile compatible with their use as attenuated vaccines (Costes et al., 2008, Fuchs et al., 2011, Ouyang et al., 2013). This list of failures illustrates the difficulties of predicting the impact of gene deletions in alloherpesviruses, particularly as there is little genetic similarity between these viruses and the much more extensively studied members of the family *Herpesviridae* (Boutier et al., 2015a).

Recently, we reported the development of an attenuated CyHV-3 vaccine candidate compatible with mass vaccination of carp (Boutier et al., 2015b). The attenuation depends on an accidentally generated deletion affecting two divergently transcribed genes (ORF56 and ORF57), removing most of ORF56 (leaving less than 10% at the 3′ end) and part of ORF57 (leaving about 75% at the 3′ end). Using *in vivo* imaging system and quantitative PCR, we demonstrated that this doubly deleted virus and the wild-type parental virus have similar tropisms. However, compared to the parental wild-type virus, the spread of the deleted virus to the other organs was much slower, and its replication was reduced in intensity and duration (Boutier et al., 2015b). The relative contributions of ORF56 and ORF57 to the safety and efficacy profile of the doubly deleted virus are not known. The functions of these ORFs are similarly not known, although ORF57 is conserved in all cypriniviruses and ORF56 is conserved only among cypriniviruses infecting cyprinid fish (Davison et al., 2013, van Beurden et al., 2010). In the present study, we investigated the relative contributions of ORF56 and ORF57 to the safety and efficacy profile of the doubly deleted virus. The results demonstrated that ORF57 encodes an essential CyHV-3 virulence factor, and raised the possibility that ORF57 orthologues in other cypriniviruses may present promising targets for rational design of attenuated vaccines.

## 2 Materials and methods

### 2.1 Cells and viruses

CCB cells (Neukirch et al., 1999) were cultured as described previously (Boutier et al., 2015b). The CyHV-3 FL and M3 strains were isolated from the kidney tissue of koi carp that died during independent CyHV-3 outbreaks in Belgium. The FL bacterial artificial chromosome plasmid (BAC) was generated from the FL strain (Costes et al., 2008). The mutant deleted for ORF56 and ORF57 (Δ56-57) was produced from this BAC (Boutier et al., 2015b).

### 2.2 Production of recombinant viruses

#### 2.2.1 Generation of recombinant BACs

**Fig. 1:**
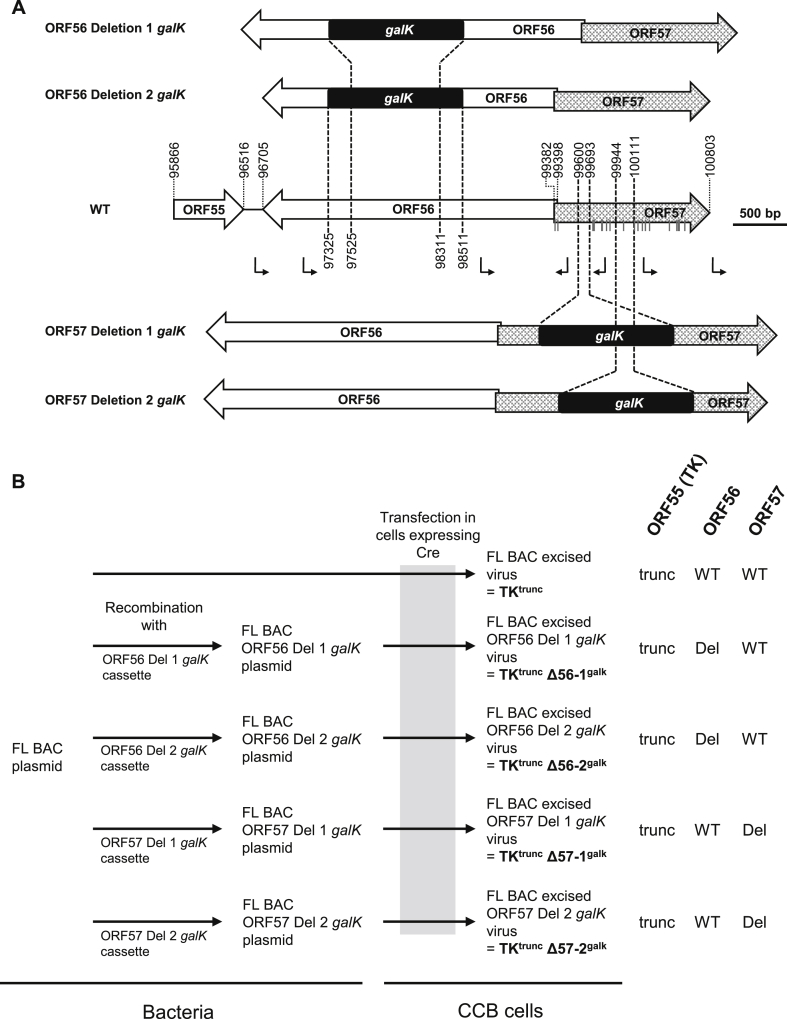
**Production of ORF56 and ORF57 *galK* singly deleted recombinants.** (A) Schematic representation of the structure of the ORF56-57 region. CyHV-3 ORFs are represented by arrows, and predicted promoters are represented by angular black arrows. In-frame ATG codons in ORF57 are indicated by dark grey vertical lines. Regions of ORF56 or ORF57 were replaced by a *galK* cassette (black rectangle) in order to preserve the predicted promoters of the non-deleted ORF. All coordinates correspond to the reference CyHV-3 sequence available in GenBank (NC_009127.1). (B) Flow chart of the production of the ORF56 and ORF57 *galK* singly deleted recombinants. Short names for the recombinants used in the text are shown in bold font. Genotypes for ORF55 (TK), ORF56 and ORF57 are shown to the right. WT, wild-type; Del, deleted; trunc, truncated.

Fig. 1, Fig. 4 outline the strategies used to generate various recombinant viruses from the FL BAC (Costes et al., 2008) on the basis of *galK* positive/negative selection in bacteria (Costes et al., 2008, Warming et al., 2005). Recombination cassettes encoding *galK* were produced by PCR, using the primers listed in Table S1 and the p*galK* vector as template. The ORF57 Rev and ORF57 NS cassettes were amplified from plasmids encoding a wild-type (57Rev) and mutated (57NS) ORF57, respectively, by using the primers listed in Table S1. Details of the ORF57 NS cassette encoding in-frame stop codons in ORF57 are given in Fig. 4A.

#### 2.2.2 Generation of recombinant viruses from BACs

Recombinant BACs were co-transfected into CCB cells with the pGEMT-TK plasmid or the pEFIN3 NLS Cre plasmid (both at a molecular ratio of 1:75) by using polyethylenimine (3 μg per 1 μg DNA) (Costes et al., 2008). Transfection with the pGEMT-TK plasmid induced recombination upstream and downstream of the BAC cassette, leading to reversion to a wild-type TK (ORF55) locus (FL BAC revertant viruses). Transfection with the pEFIN3 NLS Cre plasmid induced expression of a nuclear Cre recombinase and caused cre-loxP-mediated excision of the BAC cassette, leading to viruses expressing a truncated form of TK due to the retention of a 172 bp sequence from the BAC cassette in the TK locus (FL BAC excised viruses). Since the BAC cassette encodes the enhanced green fluorescent protein (EGFP), EGFP-negative plaques were picked and amplified in both cases. The strategies are shown in Fig. 1, Fig. 4, which also specify in bold font the short names for the recombinant viruses that are used below.

#### 2.2.3 Genetic characterization of recombinant viruses

CyHV-3 recombinants were characterized by restriction fragment length polymorphism (RFLP) analysis using SacI digestion, Southern blot analysis, sequencing of manipulated regions, and full-length genome sequencing as described previously (Boutier et al., 2015b, Costes et al., 2009, Ouyang et al., 2013, Rakus et al., 2017).

### 2.3 *In vitro* experiments

#### 2.3.1 Antibodies against the ORF56 and ORF57 proteins

Mouse polyclonal antibodies (pAbs) directed against the predicted unstructured domain (analysed by using IUPred, http://iupred.enzim.hu) of the ORF56 protein (pORF56) (specified by coordinates 98049-99398, NC_009127.1) or against the full-length ORF57 protein (pORF57) (specified by coordinates 99382-100803, NC_009127.1) were produced by DNA immunization using a commercial service (DelphiGenetics). Mouse monoclonal antibody (mAb) 6B2 was selected from a bank of mouse mAbs raised against CyHV-3 structural proteins, and recognizes an epitope in the last 165 amino acid residues of pORF57 (specified by coordinates 100309-100803, NC_009127.1).

#### 2.3.2 Indirect immunofluorescent staining

Cells were fixed in phosphate-buffered saline (PBS) containing 4% (w/v) paraformaldehyde, and permeabilized in PBS containing 0.1% (v/v) NP-40. Incubation of antibodies and washes were performed in PBS containing 10% (v/v) foetal calf serum (FCS). Mouse pAbs raised against pORF56 (diluted 1:500), mouse mAb 6B2 raised against pORF57 (diluted 1:2500), mouse pAbs raised against pORF57 (diluted 1:500), and rabbit pAbs raised against CyHV-3 structural proteins (diluted 1:1500) were used as primary antibodies (37 °C for 1 h). Alexa Fluor 488 GAM IgG(H + L) and Alexa Fluor 568 GARb IgG(H + L) (Invitrogen) were used as secondary antibodies (37 °C for 30 min). Cell nuclei were stained by using TO-PRO-3 iodide (Invitrogen, 1:1000 at room temperature for 15 min) or DAPI dilactate (Invitrogen, 1:30,000 in PBS at room temperature for 5 min). After washing, cells were mounted by using Prolong Gold antifade reagent (Invitrogen). Confocal analyses were performed by using Leica SP2 and SP5 instruments.

#### 2.3.3 Plaque size assay

Cultures of CCB cells in six-well plates were inoculated with 100–200 plaque-forming units (PFU)/well of virus for 2 h and overlaid with Dulbecco's modified essential medium (DMEM, Sigma) containing 4.5 g/L glucose, 10% (v/v) FCS and 1.2% (w/v) carboxymethylcellulose (medium viscosity, Sigma). Viral plaques were treated for indirect immunofluorescent staining by using mAb 2F12, which recognizes an unknown CyHV-3 epitope. Viral plaques were imaged by using a Nikon A1R confocal microscope, and plaque size was measured by using the ImageJ software.

#### 2.3.4 Growth curve assays

Triplicate cultures of CCB cells were infected with virus at 0.05 or 1 PFU/cell. After an incubation period of 2 h, the cells were washed with PBS and overlaid with DMEM containing 4.5 g/L glucose and 10% (v/v) FCS. The supernatant was removed from infected cultures at successive intervals (0, 2, 4 and 6 days post-infection (dpi)) and stored at −80 °C. Viral titration was carried out by duplicate plaque assays in CCB cells as described previously (Costes et al., 2009).

### 2.4 *In vivo* experiments

#### 2.4.1 Fish

Common carp were kept in 60 L tanks at 24 °C. Water parameters were checked twice per week. Microbiological, parasitic and clinical examinations carried out immediately prior to the experiments demonstrated that the fish were healthy. All experiments were preceded by an acclimation period of at least 2 weeks.

#### 2.4.2 Inoculation of fish

Two modes of inoculation were used: immersion in infectious water, and cohabitation with newly infected fish. For inoculation by immersion, fish were kept in water containing virus for 2 h under constant aeration, having adapted the volume according to fish size and fish number to utilise a biomass of approximately 10%. At the end of the incubation period, the fish were returned to their initial tank. For inoculation by cohabitation, newly infected fish were produced by immersion of naïve fish for 2 h in water containing 400 PFU/ml of the virus. At the end of the incubation period, the newly infected fish were released into the tank of fish to be infected at a ratio of 2:20.

#### 2.4.3 Ethics statement

The experiments, maintenance and care of fish complied with the guidelines of the European Convention for the Protection of Vertebrate Animals used for Experimental and other Scientific Purposes (CETS n° 123). The animal studies were approved by the local ethics committee of the University of Liège, Belgium (laboratory accreditation No. 1610008, protocol no. 1059). All efforts were made to minimize suffering.

### 2.5 Statistical analysis

Viral plaques sizes and viral growth curves were compared by two-way ANOVA with interactions followed by Bonferroni post-hoc test. Survival curves were compared by using Logrank tests. Statistical significance was represented as follows: ns, not significant; *, p < 0.05; **, p < 0.01; and ***, p < 0.001.

## 3 Results

### 3.1 Production of ORF56 and ORF57 singly deleted recombinants

To investigate the relative contributions of ORF56 and ORF57 to the attenuation reported for Δ56-57 (Boutier et al., 2015b), ORF56 and ORF57 *galK* singly deleted recombinants were produced (Fig. 1). The deletions were informed by predictions of promoter locations (made using http://fruitfly.org/seq_tools/promoter.html), in order to minimize the risk of a deletion in one gene having a polar effect on expression of the other. The strategy relied on inducing a specific deletion within the target ORF and simultaneously inserting a *galK* cassette, thereby theoretically disrupting expression of the remaining downstream sequence. The BAC cassette was removed by Cre-recombinase-mediated excision, leading to truncation of TK. As shown in Fig. 1, two independent recombinants were produced for each ORF (TK^trunc^ Δ56-1^galk^ and TK^trunc^ Δ56-2^galk^, and TK^trunc^ Δ57-1^galk^ and TK^trunc^ Δ57-2^galk^). A corresponding recombinant in which the two ORFs were intact was also made (TK^trunc^). The structures of the recombinants were confirmed by combined SacI RFLP-Southern blot analysis (Fig. S1) and full-length genome sequencing (data not shown).

**Fig. 2:**
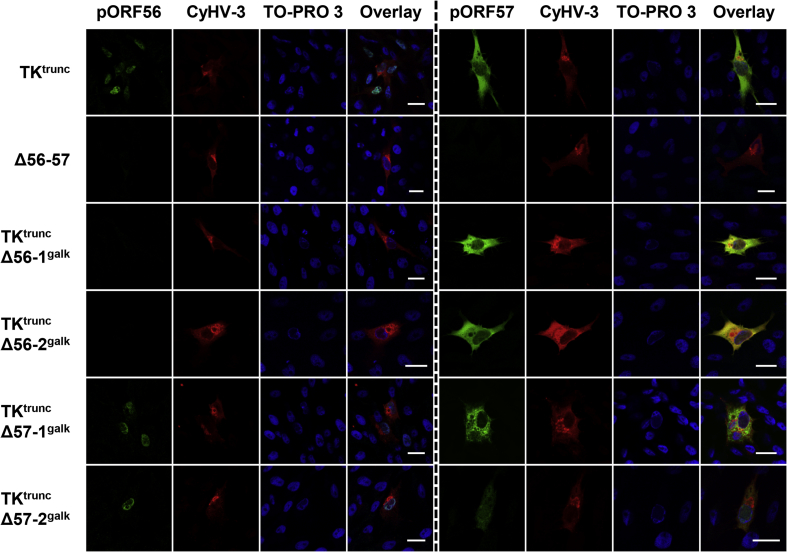
**Expression of pORF56 and pORF57 by singly deleted recombinants.** CCB cells were infected with the viruses indicated on the left at 0.05 PFU/cell. Cells were subjected at 1 dpi to indirect immunofluorescent staining as indicated at the top, detecting pORF56 (green), pORF57 (green, using mAb 6B2), CyHV-3 structural proteins (red) and DNA (TO-PRO 3). Scale bars = 20 μm.

Expression of pORF56 and pORF57 by the recombinants *in vitro* was investigated by indirect immunofluorescent staining (Fig. 2). Staining of cells infected by TK^trunc^ revealed that pORF56 and pORF57 were expressed as a nuclear protein and a cytosolic and nuclear protein, respectively. The specificity of immunostaining was demonstrated by the absence of staining in cells infected with Δ56-57. Cells infected with TK^trunc^ Δ56-1^galk^ or TK^trunc^ Δ56-2^galk^ expressed a level of pORF57 similar to that of TK^trunc^, whereas no expression of pORF56 was detected, thus demonstrating the absence of a polar effect of the ORF56 mutations on ORF57 expression. Similarly, immunostaining of cells infected with TK^trunc^ Δ57-1^galk^ or TK^trunc^ Δ57-2^galk^ revealed a pattern of pORF56 expression comparable to that of TK^trunc^, thus demonstrating the absence of polar effect of the ORF57 mutations on ORF56 expression. Staining of pORF57 revealed that both ORF57-mutated viruses expressed at least part of the remaining sequence of the ORF (Fig. 2). However, the level of expression of truncated protein was much lower than that observed for intact pORF57 in TK^trunc^, being barely detectable for TK^trunc^ Δ57-2^galk^. Also, the distribution pattern was affected, with the intact protein expressed as a diffuse cytosolic protein and the truncated form localized in cytosolic agglutinates. The results of this experiment demonstrated that the ORF56 and ORF57 *galK* singly deleted recombinants were appropriate for testing the relative contribution of these two genes to the safety profile of Δ56-57.

### 3.2 Effect of deleting ORF56 or ORF57 on CyHV-3 virulence *in vivo*

**Fig. 3:**
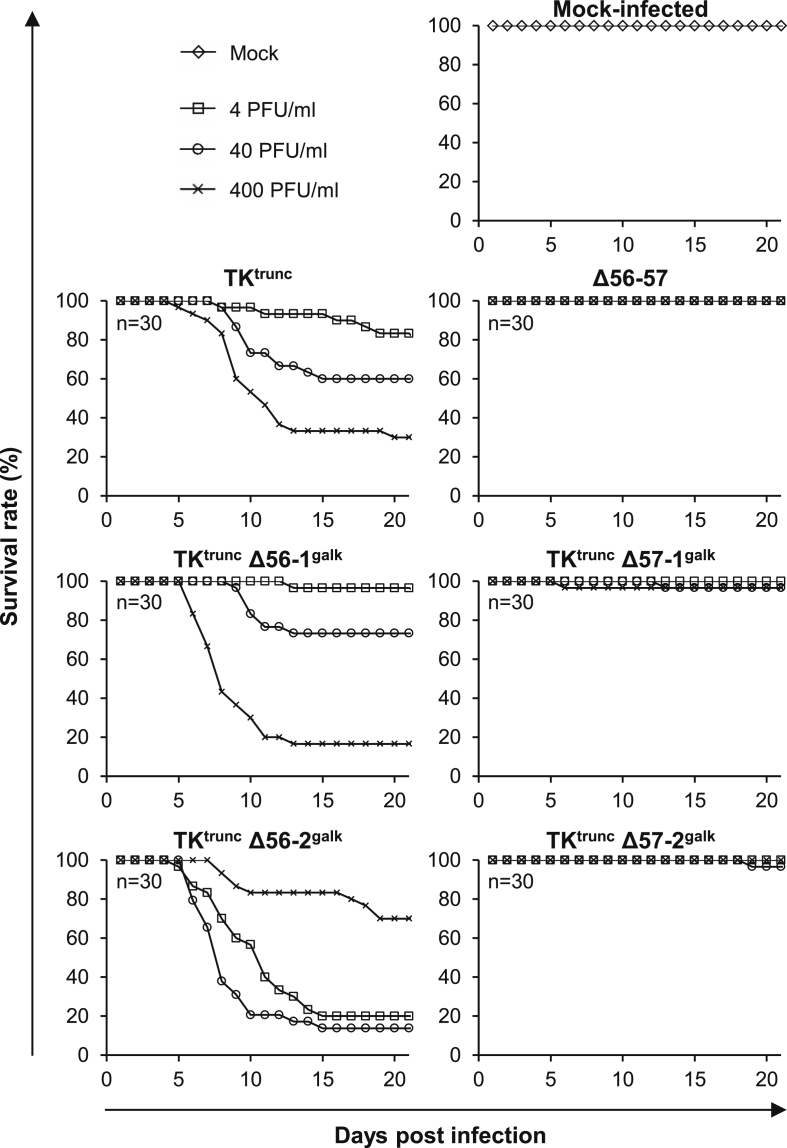
**Effect of ORF56 and ORF57 deletion on CyHV-3 virulence.** The virulence of the indicated recombinants was tested in carp (n = 30, average weight 3.77 g ± 1.95 g, 7 months old). Fish were infected with the indicated viruses by immersion for 2 h in water containing 4 (□), 40 (○), or 400 (x) PFU/ml, or mock infected (◊). Data are presented as survival rates expressed in relation to the number of fish at time of inoculation.

Fish were inoculated with the ORF56 or ORF57 *galK* singly deleted recombinants, or with TK^trunc^ and Δ56-57 as controls for virulence and attenuation, respectively, by immersion in water containing virus (Fig. 3). An additional group of fish was mock-infected.

**Fig. 4:**
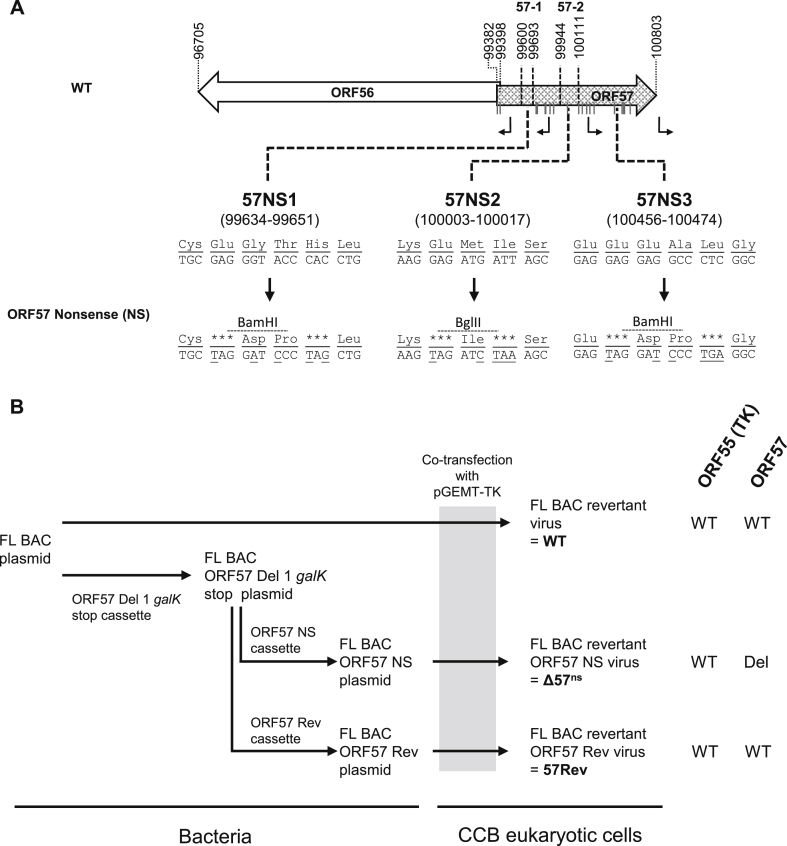
**Production of ORF57 nonsense and revertant recombinants.** (A) Schematic representation of the structures of ORF57 nonsense loci. CyHV-3 ORFs are represented by arrows, and predicted promoters in the ORF57 region are represented by angular black arrows. In-frame ATG codons in ORF57 are indicated by dark grey vertical lines. Three regions of ORF57 were mutated to create two in-frame stop codons and one novel restriction site in each. Mutated nucleotides are underlined, and stop codons are shown by asterisks. All coordinates correspond to the reference CyHV-3 sequence available in GenBank (NC_009127.1). (B) Flow chart of the production of ORF57 nonsense and revertant recombinants. Short names for the recombinants used in the text are shown in bold font. Genotypes for ORF55 (TK) and ORF57 are shown to the right. WT, wild-type; Del, deleted.

Fish infected with TK^trunc^ exhibited all the clinical signs associated with CyHV-3 disease, including apathy, folding of the dorsal fin, hyperemia, increased mucus secretion, skin lesions, suffocation, erratic swimming and loss of equilibrium. Fish infected with TK^trunc^ Δ56-1^galk^ or TK^trunc^ Δ56-2^galk^ also expressed severe clinical signs, and survival rates were comparable to, or lower than, that of fish infected with TK^trunc^ (p = 0.81 (ns) and p < 0.05 (*), respectively). In contrast, fish infected with TK^trunc^ Δ57-1^galk^ or TK^trunc^ Δ57-2^galk^ expressed mild disease, and survival rates were comparable to that observed for fish infected with Δ56-57 (p = 0.16 (ns) and p = 0.32 (ns), respectively). However, although clinical signs were very limited in fish infected with Δ56-57 (a few exhibiting folding of the dorsal fin for a day or two), infection with TK^trunc^ Δ57-1^galk^ or TK^trunc^ Δ57-2^galk^ led to obvious clinical signs for few days in all fish, including folding of the dorsal fin and skin hyperemia. These results revealed that TK^trunc^ Δ57-1^galk^ and TK^trunc^ Δ57-2^*galk*^ are less attenuated than Δ56-57. This weaker attenuation could be due to expression of pORF57 as a truncated protein, or to a contribution of the ORF56 deletion to the attenuated phenotype observed for Δ56-57. Nonetheless, these experiments demonstrated that ORF57 is a key CyHV-3 virulence factor.

### 3.3 Production of an ORF57 recombinant virus encoding multiple stop codons

To investigate further the role of ORF57 as a virulence factor and to test the potential of a recombinant specifically lacking ORF57 as a vaccine candidate, an ORF57 nonsense mutant containing a wild-type TK locus was produced (Δ57^ns^; Fig. 4). Point mutations were made at three separate locations in ORF57 in order to create in-frame stop codons and novel restriction sites (57NS1-3; Fig. 4A). A wild-type recombinant (WT) and a recombinant in which the mutated ORF57 was reverted to wild-type were also produced (57Rev) (Fig. 4B). The structures of the recombinants were confirmed by RFLP, Southern blot analysis (Fig. S2A), a combined PCR-restriction digestion approach (Fig. S2B) and full length genome sequencing (data not shown).

**Fig. 5:**
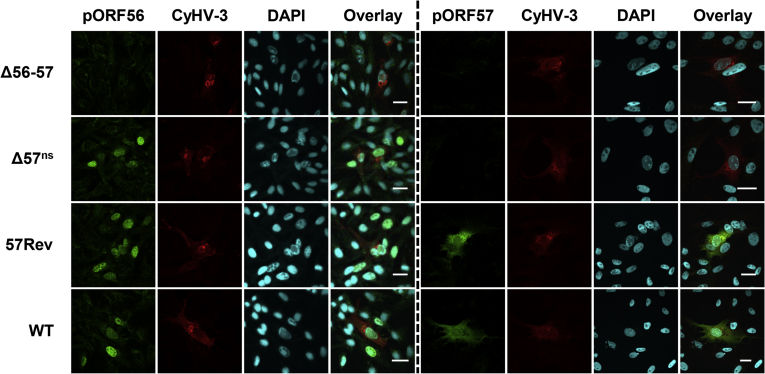
**Expression of pORF56 and pORF57 by CyHV-3 ORF57 nonsense and revertant recombinants.** CCB cells were infected with the viruses indicated on the left at 0.05 PFU/cell. Cells were subjected at 1 dpi to indirect immunofluorescent staining as indicated at the top, detecting pORF56 (green), pORF57 (green, using a pAb against pORF57), CyHV-3 structural proteins (red) and DNA (DAPI). Scale bars = 20 μm.

Expression of pORF56 and pORF57 by these recombinants was investigated by immunofluorescent staining (Fig. 5). Staining of pORF56 revealed similar expression by Δ57^ns^, 57Rev or WT. Staining of pORF57 was performed with a pAb raised against the entire protein in order to reveal the production of truncated forms potentially expressed by Δ57^ns^ (note that pORF57 staining in Fig. 2 was performed by using a mAb) (Fig. 5). Immunostaining of cells infected by WT and 57Rev revealed expression of pORF57, whereas no staining was observed in cells infected with Δ56-57 or Δ57^ns^ (Fig. 5). These experiments demonstrated that Δ57^ns^ and 57Rev were appropriate for testing the relative contributions of ORF57 to the safety and efficacy profile of Δ56-57 as a candidate vaccine.

### 3.4 Effect of ORF57 deletion on CyHV-3 growth *in vitro*

**Fig. 6:**
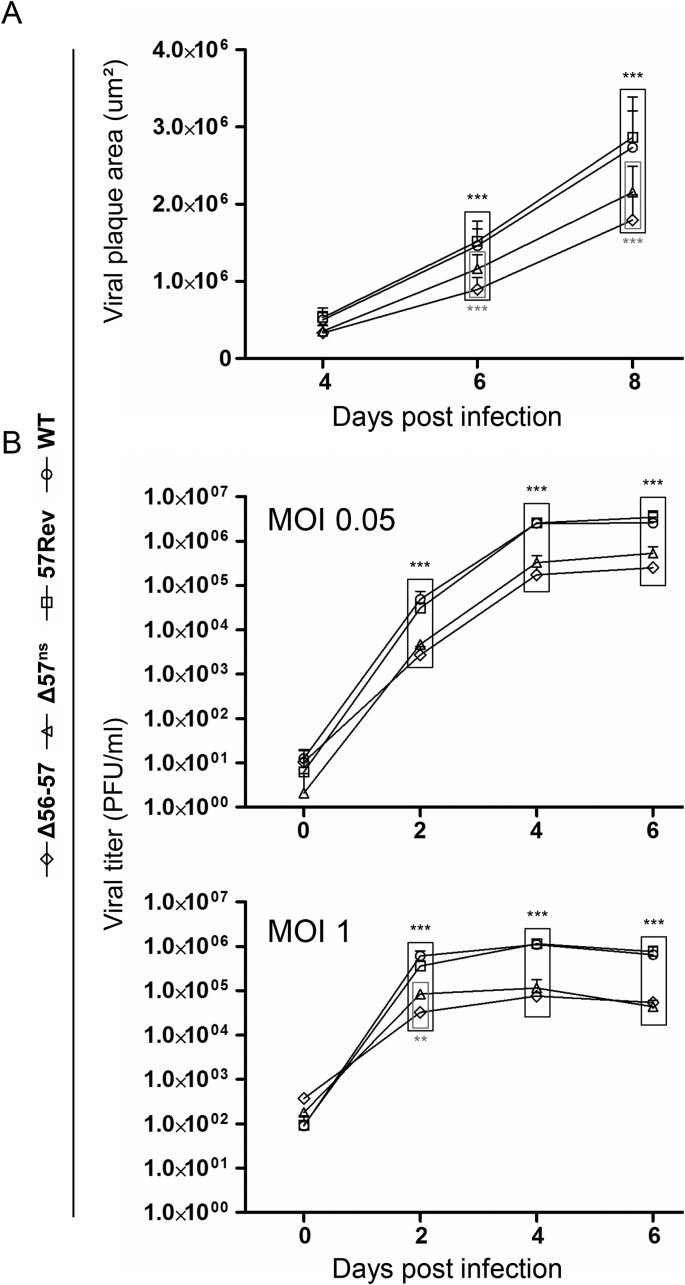
**Effect of ORF57 deletion on CyHV-3 replication *in vitro*.** (A) Viral plaque assay. Data presented are the mean + SD of 30 measurements. (B) Viral growth assay. Data presented are the mean + SD of triplicate measurements. Significant differences between WT or 57Rev and Δ56-57 or Δ57^ns^ are shown in black, and significant differences between Δ56-57 and Δ57^ns^ are shown in grey.

The Δ56-57 has been shown to have a growth defect *in vitro* (Boutier et al., 2015b). Δ57^ns^ was used to determine the relative contribution of the loss of ORF57 function to this defect. Both Δ56-57 and Δ57^ns^ expressed significantly smaller plaques than WT and 57Rev (p < 0.001 (***)) (Fig. 6A), and Δ57^ns^ expressed plaques that were significantly larger than those of Δ56-57 (p < 0.001 (***)). Viral replication *in vitro* was also investigated by growth curve assay at 0.05 and 1 PFU/cell (Fig. 6B). Consistent with earlier observations (Boutier et al., 2015b), and independent of the multiplicity of infection used, Δ56-57 produced fewer infectious particles than WT and 57Rev (p < 0.001 (***)). Similarly, Δ57^ns^ produced fewer infectious particles (p < 0.001 (***)) than these two control viruses. These experiments showed that loss of ORF57 function contributes greatly to the viral replication defect observed for Δ56-57.

### 3.5 Testing of an ORF57 deleted recombinant as an attenuated vaccine candidate against CyHV-3

**Fig. 7:**
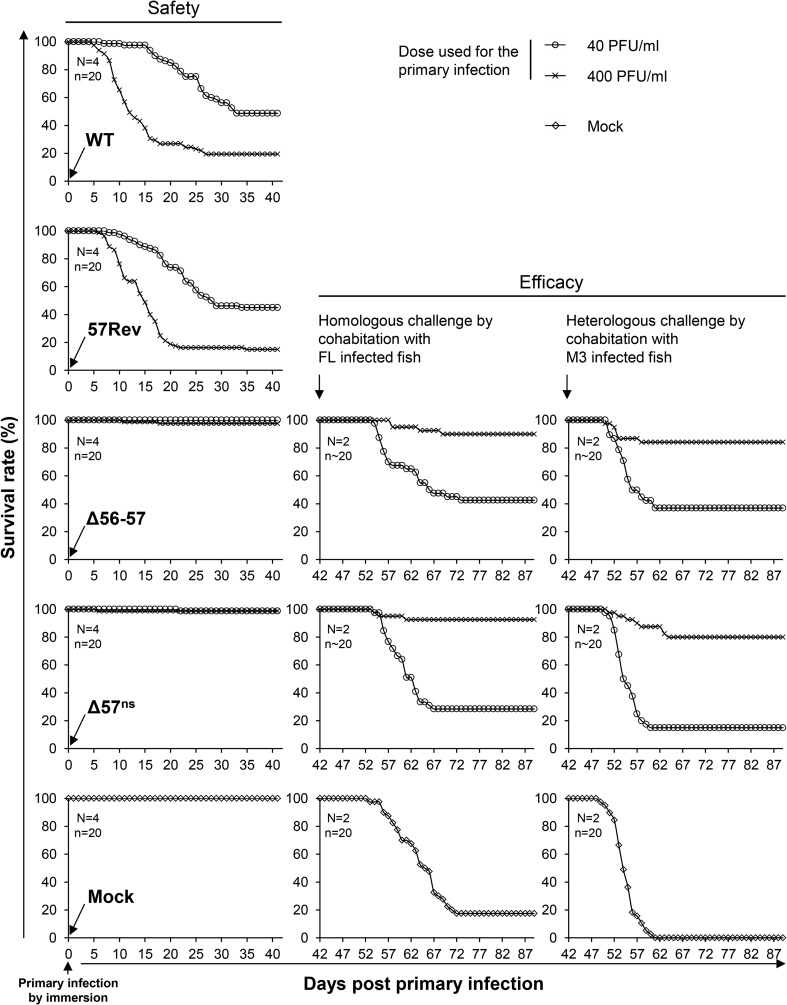
**Effect *in vivo* of nonsense mutations in ORF57.** In the panels on the left, the level of attenuation (safety) of Δ57^ns^ was tested in carp. WT and 57 Rev were used as positive controls for virulence, and Δ56-57 was used as a control for attenuation. Fish (a total of 80 fish distributed in 4 tanks, average weight 2.42 ± 0.95 g, 6 months old) were infected by immersion for 2 h in water containing 40 (○) or 400 (x) PFU/ml of virus, or were mock infected (◊). Immune protection induced by the primary infection (efficacy) was assessed at 42 dpi. Two of the four tanks initially infected with Δ56-57 or Δ57^ns^, or mock-infected, were challenged by cohabitation with wild-type strain FL-infected fish (middle column), and the two others were challenged by cohabitation with wild-type strain M3-infected fish (right column). Data are presented as mean survival rate (safety, mean of 4 tanks; efficacy, mean of 2 tanks) expressed according to the number of fish at the time of primary infection (safety) or challenge (efficacy).

Fish were inoculated at two different doses with Δ57^ns^, 57Rev, WT or Δ56-57 by immersion in water containing the virus (Fig. 7), or were mock-infected. Fish infected with 57Rev expressed severe clinical signs, and the survival curves were comparable to those for WT (p = 0.18 and 0.74 (ns) at 40 and 400 PFU/ml, respectively) (Fig. 7). For both WT and 57Rev, one of the four tanks infected at 40 PFU/ml did not show any clinical signs and mortalities, suggesting an unusually low susceptibility of fish at the time of infection. Fish infected by Δ57^ns^ expressed mild symptoms but survival rates were comparable to those observed with Δ56-57 (p = 0.32 and 0.57 (ns) at 40 and 400 PFU/ml, respectively). Survival rates following Δ57^ns^ and Δ56-57 infection were similar to those of mock-infected groups (p = 0.32 and 0.16 (ns), respectively, at 400 PFU/ml). These results further demonstrate the role of ORF57 as a key virulence factor.

To assess the level of immune protection conferred by primary infection with Δ57^ns^ or Δ56-57, fish infected with these viruses were challenged by cohabitation with carp infected by the WT FL or M3 strains (two tanks per strain). Fish that were mock-infected at the time of the primary infection were used as a negative control (Fig. 7). All of the mock-infected fish succumbed to challenge with the M3 strain, and fewer than 20% survived challenge with the FL strain. Primary infection with Δ56-57 or Δ57^ns^ induced comparable levels of protection in a dose-dependent manner. After primary infection at 400 PFU/ml with either virus, similar levels of relative percentage survival were observed (about 80% and 90% for the M3 and FL strains, respectively). These results suggest that Δ56-57 and Δ57^ns^ induced similar levels of protection against homologous or heterologous challenge.

## 4 Discussion

The contributions of ORF56 and ORF57 to the safety and efficacy profile of the Δ56-57 attenuated vaccine candidate were addressed by producing several recombinants and analysing them *in vitro* and *in vivo*. The results demonstrated that ORF57, in contrast to ORF56, is an essential CyHV-3 virulence factor.

The safety and efficacy profile of a recombinant containing multiple nonsense mutations in ORF57 (Δ57^ns^) was comparable to that of Δ56-57 (Fig. 7). The more severe clinical signs observed following infection with TK^trunc^ Δ57-1^galk^ or TK^trunc^ Δ57-2^galk^, compared to those observed with Δ57^ns^, suggest that the truncated ORF57 proteins expressed by these mutants may retain partial function (Fig. 2, Fig. 5). In contrast, expression of truncated proteins by Δ56-57 was not detected, even though this virus retains the major part of ORF57 (Fig. 2, Fig. 5). The immune protection conferred by the Δ57^ns^ was comparable to that of Δ56-57 (Fig. 7). Our former study on the development of the Δ56-57 virus as an attenuated recombinant vaccine candidate demonstrated that it confers a mucosal immunity at the portal of entry (Boutier et al., 2015b). Further studies are required to determine whether the Δ57^ns^ confers also this type of protection and to establish correlates of protection/immunity associated with long-term protection against CyHV-3 disease. It is already known that anti-CyHV-3 antibodies do not represent correlates of protection (Perelberg et al., 2008). Indeed, as early as 280 dpi, the titer of anti-CyHV-3 antibodies in previously infected fish is only slightly higher or comparable to that of naive fish. Nevertheless, immunized fish, even those in which antibodies are no longer detectable, are resistant to a lethal challenge, possibly because of the subsequent rapid response of B and T memory cells to antigen restimulation (Perelberg et al., 2008).

ORF57 is conserved among the cypriniviruses, which currently comprise CyHV-1, CyHV-2, CyHV-3 and AngHV-1, with the likelihood of an expanded list in future (Doszpoly et al., 2015). Amino acid sequence similarity in ORF57 ranges from 67.5 to 72.4% among the three cyprinid herpesviruses, and from 57.9% to 60.4% between these viruses and AngHV-1 (Table S2). A homologue is also present in a virus in a different family, crocodile poxvirus (CrPV) (Aoki et al., 2007, Davison et al., 2013, van Beurden et al., 2010, Waltzek et al., 2009), suggesting that horizontal gene transfer may have occurred. The present study suggests that ORF57 orthologues in other pathogenic viruses in the genus *Cyprinivirus* may offer promising targets for the design of attenuated recombinant vaccines.

To our knowledge, no studies have been reported on the role of any ORF57 orthologue, and extensive bioinformatic analyses have not shed any light on possible functions. CyHV-3 pORF57 is known to be abundant in virus particles (Michel et al., 2010, van Beurden et al., 2011), and its orthologue in AngHV-1 (pORF35) has been described as an abundant constituent of the virion tegument (van Beurden et al., 2011). This property is consistent with the diffuse, mainly cytoplasmic expression pattern of pORF57 in CyHV-3-infected cells (Fig. 2, Fig. 5). Δ57^ns^ will be a valuable tool in unravelling the roles of ORF57 in the replication cycle of CyHV-3 *in vitro* and *in vivo*.

Aquaculture is the fastest growing food production sector in the world. Despite the devastating losses caused by alloherpesviruses, no commercial vaccines are available to control these pathogens. The present study is the first to identify an essential virulence factor in one of these viruses. The presence of an orthologue in all other cypriniviruses opens new possibilities for the development of prophylactic vaccines against this group of economically important viruses.

## Conflicts of interest

The authors declare no conflict of interest.
