# Supplementary material for: Identification of an essential virulence gene of cyprinid herpesvirus 3
Source: Antiviral Res. 2017 Sep;145:60–9. doi: 10.1016/j.antiviral.2017.07.002 (PMC5588920; doi:10.1016/j.antiviral.2017.07.002)
Supplement: Supplementary file 1 [file mmc1.docx]

**SUPPORTING INFORMATION**

**SUPPLEMENTARY FIGURES**

**
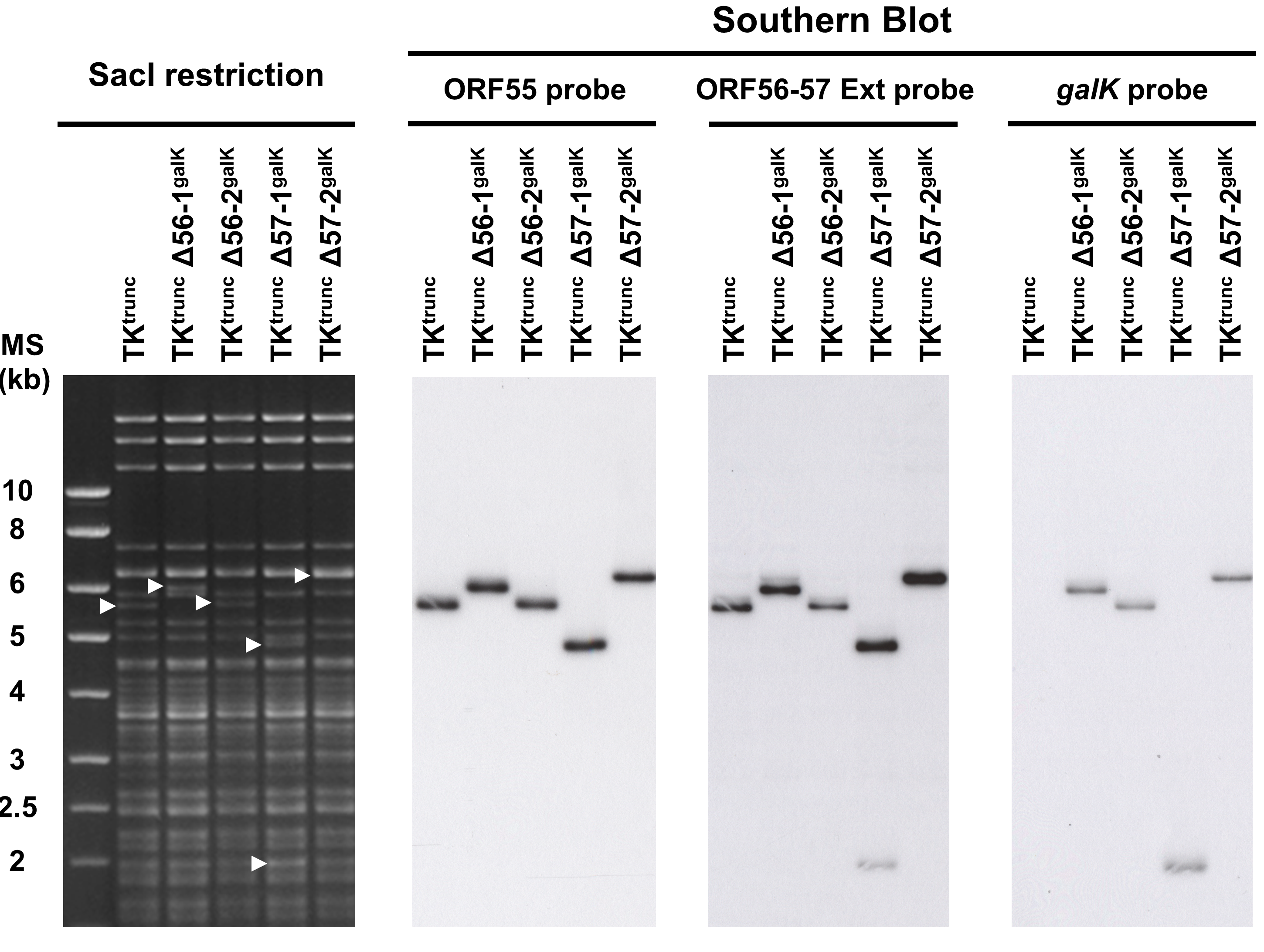
**

**Fig. S1. Structural analysis of ORF56 and ORF57 *galK* singly deleted recombinants.** The viruses indicated were analysed by SacI restriction (left) and Southern blotting (right) using ORF55, ORF56‑57 External (Ext) and *galK* probes. White arrowheads indicate fragments containing ORF56‑57. Markers sizes (MS) are indicated on the left.

**
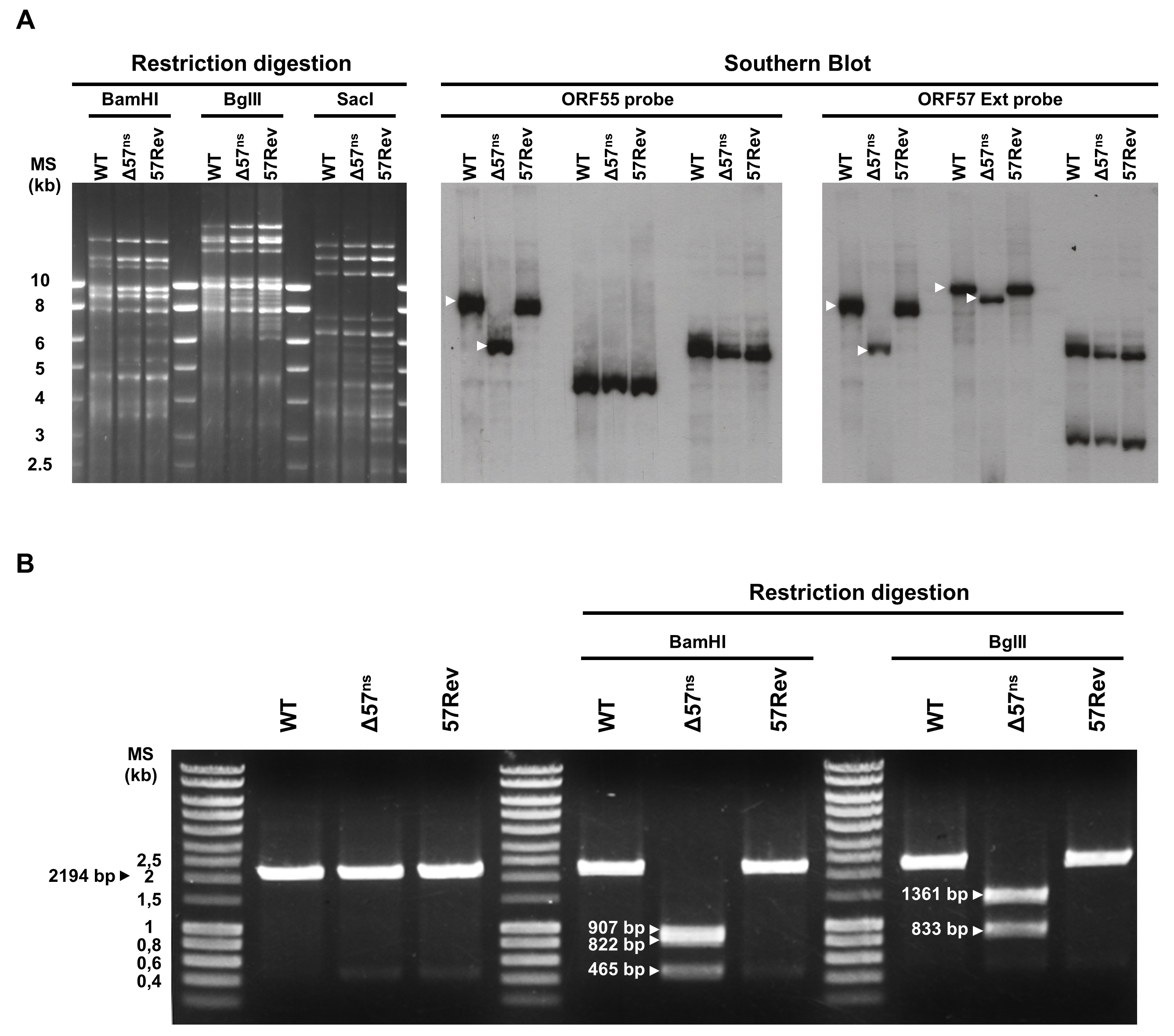
**

**Fig. S2. Structural analysis of ORF57 nonsense and revertant recombinants. (A)** The viruses indicated were analysed by SacI, BglII and BamHI restriction (left) and Southern blotting using ORF55 and ORF57 External (Ext) probes. White arrowheads indicate fragments containing the ORF57 locus that are modified following insertion of new BglII and BamHI sites in Δ57^ns^. Marker sizes (MS) are indicated on the left. **(B)** PCR amplification of ORF57 (left panel) followed by restriction digestion with BamHI and BglII. The predicted sizes of intact and digested amplicons are indicated. Marker sizes (MS) are indicated on the left.

**SUPPLEMENTARY TABLES**

**Table S1. Primers**

| Probe or cassette name | Primer name | Sequence (5’- 3’) | Coordinates* |
| --- | --- | --- | --- |
|  |  |  |  |
| **Probes for Southern blot analysis** | | | |
| CyHV-3 ORF55 | ORF55InF | AGCGCTACACCGAAGAGTCC | 95990-96009 |
|  | ORF55stopR | TCACAGGATAGATATGTTACAAG | 96516-96494 |
| CyHV-3 ORF 56-57 Del | ORF56-57Pr5F | GGTACAAGACGGCCTGCTG | 97247-97265 |
|  | ORF56-57Pr9R | GCCAGCACGTAGAGCTTGTG | 99686-99667 |
| CyHV-3 ORF56-57 Ext | ORF56-57Pr4F | AGCCCGTGTGTCCGTACTTT | 96734-96753 |
|  | ORF57In2R | GGGCTCTTTGACGGAGTACA | 100596-100577 |
| CyHV-3 ORF57 Ext | ORF56-57Pr9F | TCTTCCTCGGTCCGTTTACC | 99175-99194 |
|  | ORF58InR | GTGGGAAGGAGGGAGGTGAG | 101349-101368 |
| *galK* | *galK*F2 | AGGTGAGGAACTAAACCCAG |  |
|  | *galK*R2 | GATAAAGCTGCTGCAATACG |  |
| **Recombination cassettes** | | | |
| ORF 56 Del 1 *galK* | ORF56-1 *galK* F | TCAGGATCGAGGTCACCAGCTTGAGCTTCTCGGGCATGTACTCGCGCCACCCTGTTGACAATTAATCATCGGCA | 97475-97524 |
|  | ORF56-1 *galK* R | CGGCGAGGTGATTTCGGTCATGAGCAAATCGATTGCGGCCGAACAGCAGCTCAGCACTGTCCTGCTCCTT | 98361-98312 |
| ORF 56 Del 2 *galK* | ORF56-2 *galK* F | GATCGGGTACGTCGGCGTGCGCCACTTGACCTTCCTCAACGTCCCCGTCACCTGTTGACAATTAATCATCGGCA | 97275-97324 |
|  | ORF56-2 *galK* R | GCGCACACCATCACCATCTGTCCCATGTCTCCCCAACGCTACACCGTGACTCAGCACTGTCCTGCTCCTT | 98561-98512 |
| ORF 57 Del 1 *galK* | ORF57-1 *galK* F | CGTACAGGGTGGCGGTGCACCTGTCCCAGAAGGCCTTCACCGCCTGG*GAGCTC*CCTGTTGACAATTAATCATCGGCA | 99551-99599 |
|  | ORF57-1 *galK* R | CGGCTCATCATCTGCGGGTCCATCCAGGCGCCCTTGCCCCACAGCAGAGC**T**TCAGCACTGTCCTGCTCCTT | 99743-99694 |
| ORF 57 Del 2 *galK* | ORF57-2 *galK* F | CTTTGTGCTGCACAAGGGCTTCAACCACCACTACGCCTTCTGCGATCACCCCTGTTGACAATTAATCATCGGCA | 99894-99943 |
|  | ORF57-2 *galK* R | CTGAGCGTTGTTGAAGGCCTCCATCAGGTGCTGCCTGATCTGCTTGTGCA*GAGCTC*AGCACTGTCCTGCTCCTT | 100161-100112 |
| ORF57 Del 1 *galK* stop | ORF57-1 *galK* F | CGTACAGGGTGGCGGTGCACCTGTCCCAGAAGGCCTTCACCGCCTGG*GAGCTC*CCTGTTGACAATTAATCATCGGCA | 99551-99599 |
|  | ORF57-stop *galK* R | AACAATACTAGACTCTGACTTTTTTTATTGGTGAGTGAGTGAGCGAGATGTCAGCACTGTCCTGCTCCTT | 100804-100853 |
| ORF57 NS/Rev cassette | ORF56-57Pr9F | TCTTCCTCGGTCCGTTTACC | 99175-99194 |
|  | ORF58InR | GTGGGAAGGAGGGAGGTGAG | 101349-101368 |

* GenBank accession number NC_009127.1

Underlined: 50 bp corresponding to CyHV-3 sequence

Red: sequence corresponding to *galK*

Blue: sequence inserted to create a SacI site

Bold: sequence inserted to disrupt an existing SacI site

Italic: SacI site

**Table S2. Pairwise amino acid sequence relationships between ORF57 orthologues.**

|  | CyHV-3 ORF57  473 aa |  |  |  |  |
| --- | --- | --- | --- | --- | --- |
| CyHV-3 ORF57  473 aa |  | CyHV-2 ORF57  578 aa |  |  |  |
| CyHV-2 ORF57  578 aa | %_Id : 54.1%  %_Sim : 72.4%  Overlap : 427 aa |  | CyHV-1 ORF57  453 aa |  |  |
| CyHV-1 ORF57  453 aa | %_Id : 49.7%  %_Sim : 71.9%  Overlap : 449 aa | %_Id : 49.1%  %_Sim : 67.5%  Overlap : 434 aa |  | AngHV-1 ORF35  296 aa |  |
| AngHV-1 ORF35  296 aa | %_Id : 32.8%  %_Sim : 60.4%  Overlap : 265 aa | %_Id : 30.0%  %_Sim : 58.4%  Overlap : 267 aa | %_Id : 33.1%  %_Sim : 57.9%  Overlap : 266 aa |  | CrPV CRV155  226 aa |
| CrPV CRV155  226 aa | %_Id : 25.5%  %_Sim : 52.9%  Overlap : 208 aa | %_Id : 29.7%  %_Sim : 50.3%  Overlap : 165 aa | %_Id : 26.1%  %_Sim : 51.7%  Overlap : 203 aa | %_Id : 33.5%  %_Sim : 57.3%  Overlap : 206 aa |  |

Dual analyses of pORF57 orthologue sequences using http://fasta.bioch.virginia.edu/fasta_www2/fasta_list2.shtml

%_Id, percentage identity; %_Sim, percentage similarity; Overlap, alignment length used for the calculation; aa, amino acid residues
